
JOURNAL INFORMATION
==============================
NLM Title Abbreviation: ScientificWorldJournal
Journal ID: ScientificWorldJournal
NLM Journal ID: 101131163
Journal ID: TSWJ

The Scientific World Journal

ISSN: 2356-6140
EISSN: 1537-744X
Publisher: Wiley

ARTICLE INFORMATION
==============================
PMCID: PMC6084194
PMID: 30147597
DOI: 10.1100/tsw.2001.215
Article ID: 484687
Article version: 1
Subjects: Short Report

Semisynthesis of Protein variants Using Intein-Mediated Protein Ligation

Arnold Ulrich *
Hinderaker Matthew P.
Raines Ronald T.
Department of Biochemistry, University of Wisconsin-Madison, 433 Babcock Drive, Madison, WI 53706, USA
*Ulrich Arnold: uarnold@biochem.wisc.edu
Publication date: 2001
Electronic publication date: 2001 Nov 2
Volume: 1
First page: 117
Last page: 117
Copyright: Copyright © 2001 Ulrich Arnold et al.
Copyright year: 2001
License: This is an open access article distributed under the Creative Commons Attribution License, which permits unrestricted use, distribution, and reproduction in any medium, provided the original work is properly cited.
License URL: https://creativecommons.org/licenses/by/3.0/

Funding: National Institutes of Health GM44783

==============================
Miami *Nature Biotechnology* Short Reports
*TheScientificWorld* (2001) 1 (S3), 117SR
ISSN 1532-2246; DOI 10.1100/tsw.2001.215

### SEMISYNTHESIS OF PROTEIN VARIANTS USING INTEIN-MEDIATED PROTEIN LIGATION

Ulrich Arnold*, Matthew P. Hinderaker, and Ronald T. Raines

Department of Biochemistry, University of Wisconsin-Madison, 433 Babcock Drive, Madison, WI 53706, U.S.A.
* uarnold@biochem.wisc.edu

**INTRODUCTION.** The introduction of non-natural amino acid residues or modules into proteins enables the study of the significance of single amino acid residues or entire structural regions in the stability of the protein, its folding/unfolding behavior, or its biological function. Bovine pancreatic ribonuclease A (RNase A) is a small protein with a well-characterized structure and thoroughly studied stability and unfolding/folding behavior (1). More recently, RNase A also became a target of studies as a chemotherapeutic (2). We exploited the method of intein-mediated protein ligation (3) to introduce non-natural amino acid residues into the C-terminal part of RNase A and investigated the biochemical properties of the variants.

**METHOD.** Peptides containing either the wild-type RNase A sequence Cys95-Val124 or the same sequence with replacements for certain amino acid residues were synthesized by solid-phase methods. Full-length RNase A or RNase A 1-94 and variants of these were produced as a fusion protein. The respective DNA sequence was inserted into the plasmids pTYB1 and pTXB1 to produce a fusion protein, which consists of RNase A (1-124 or 1-94) with an additional Met –1, the intein, and a chitin-binding domain (4).

**RESULTS.** With pTYB1, purification of the fusion protein and thiol-facilitated formation of a thioester at the C-terminal Asn94 of the RNase A 1-94 fragment resulted in high amounts of cleavage product. However, only a little fraction contained the required thioester due to the slow intein cleavage and the fast thioester hydrolysis. Ligation of the intein cleavage product with the corresponding peptide yielded about 70 μg of ligation product per liter of bacterial culture. An N94A RNase A 1-94 variant proved to result in considerable higher yields of thioester, enabling efficient ligation of the protein component with the peptide. Here, ligation yielded about 200 μg of product per liter of bacterial culture. With pTXB1, the yields of fusion protein were substantially higher which allowed a formation of ligation product in reasonable amounts.

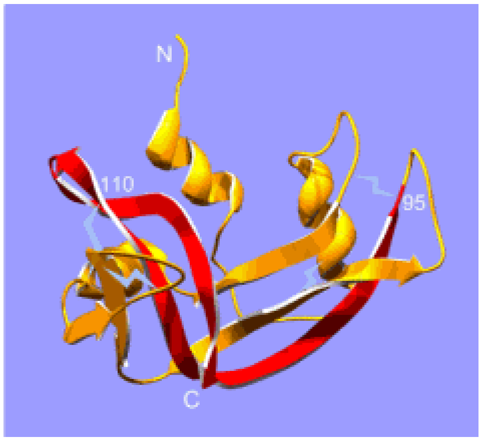

**Fig. 1.** RNase A construct: residues 1-94 were produced by use of the intein, residues 95-124 were synthesized by solid phase methods.

For comparison to semisynthetic RNase A variants with non-natural residues, wild-type RNase A 1-124 and N94A RNase A were produced by the intein method. Both intein cleavage products could be isolated at about 500 µg per liter bacterial culture.

**DISCUSSION.** Our results show that semisynthesis of RNase A variants is possible by producing a protein fragment with a C-terminal thioester and subsequent ligation to a peptide with an N-terminal Cys residue. By this means, non-natural amino acid residues and even modules can be incorporated into protein structures. Variants containing the wild type sequence of RNase A in the peptide were identical with those RNase A variants produced as full-length proteins.

**ACKNOWLEDGEMENT.** This work was supported by the Arthur B. Michael Fund and grant GM44783 (NIH).

**REFERENCES.**

1. D'Alessio, G. and Riordan, J.F. (1997) Ribonucleases: Structures and Functions. Academic Press, New York
2. Leland, P.A., Schultz, L.W., Kim, B.-M., and Raines, R.T. (1998) Proc. Natl. Acad. Sci. U S A 95, 10407-10412
3. Evans, T.C. Jr., Benner, J., and Xu, M.Q. (1998) Protein Sci. 11, 2256-2264
4. New England Biolabs: IMPACT ™ T7: One-step protein purification system
